# Polysubstance Use Disorder After Sleeve Gastrectomy

**DOI:** 10.7759/cureus.4388

**Published:** 2019-04-05

**Authors:** Yahia Albobali, Mahmoud Y Madi

**Affiliations:** 1 Psychiatry, Hamad Medical Corporation, Doha, QAT; 2 Internal Medicine, Tufts Medical Center, Boston, USA

**Keywords:** cannabis, psychosis, polysubstance abuse, amphetamines, bariatric surgery

## Abstract

We describe a case of polysubstance use disorder that occurred after sleeve gastrectomy. Alcohol, cannabis, and stimulant use disorder was diagnosed a few years after the bariatric surgery when the patient developed a substance-induced psychotic disorder. Treatments included psychotropic medications to treat his psychosis and involvement in a drug rehabilitation and relapse prevention program. This case highlights the importance of a preoperative assessment of substance use risk in patients undergoing bariatric surgeries as well as the need for close follow-up postoperatively.

## Introduction

The prevalence of obesity has been on the rise over the last few decades. Bariatric surgeries remain one of the most effective solutions to manage morbid obesity; reducing weight remarkably and improving obesity-related comorbidities. There may be related pathophysiological mechanisms underlying both addictive behaviors and overeating [1]. There is evidence that the incidence of alcohol use disorder (AUD) may increase after bariatric surgery [2]. There also seems to be a differential effect on the AUD incidence depending on the type of bariatric surgery. For example, King et al. reported that the AUD incidence was two times higher after a Roux-en-Y gastric bypass as compared to a laparoscopic adjustable gastric band [3]. Few studies have examined non-alcohol substance use disorder after bariatric surgeries. There appears to be enough evidence to suggest that a certain group of patients start using illicit drugs after undergoing such surgeries. However, a lot of these patients have had a preoperative history of substance use disorders [4].

## Case presentation

A 14-year-old boy with morbid obesity and no known prior psychiatric history underwent sleeve gastrectomy. Prior to the surgery, he weighed 167 kilograms with a body mass index (BMI) of 54.5. Within a few months postoperatively, he weighed 70 kilograms with a BMI of 22.8. The patient’s substance use disorder started at the age of 15, one year after the bariatric surgery. Of note, the patient's parents were separated and he lived with his mother and siblings. None of his family members or relatives had a history of substance use disorder. He initially started using fenethylline (marketed under the brand name Captagon), as it was a common substance used by his peers at school. He started with two tablets daily and increased his use gradually up to 15 tablets daily. He started smoking cannabis a year later, at the age of 16, starting with one cigarette per day and increasing his use gradually until reaching a peak of 20 cigarettes per day. The patient started drinking alcohol occasionally at the age of 16 as well, and it soon became an issue of excessive use on a daily basis. The patient drank different types of alcoholic beverages. He reported incidents of fainting in relation to alcohol use but had never experienced withdrawal. He mentioned that he started using alcohol as a way to reduce his use of other substances. Two years later, at the age of 18, the patient started using methamphetamine, which caused him to develop paranoid ideation, auditory hallucinations, severe insomnia, and aggressive behavior. The patient was admitted to an inpatient psychiatric unit for a few days and was started on haloperidol 3 mg orally twice daily, benztropine 2 mg orally twice daily, and quetiapine 50 mg orally as needed for insomnia. His psychotic disorder improved with the cessation of substance use and the treatments initiated on the inpatient side. After his discharge, he unfortunately relapsed and continued to use the aforementioned substances.

After arranging for close follow-up, the patient voluntarily presented to the rehabilitation center, motivated to stop using all substances, as he was legally and financially burdened by this disorder. He was incarcerated twice for substance use-related criminal charges. He was also motivated to start a new life and to enroll again in higher education, as he dropped out of school previously due to his polysubstance use disorder. The patient has thereafter been involved in a rehabilitation and relapse prevention program, which included inpatient admissions as needed to the rehabilitation center, involvement in individual and group therapy, occupational therapy, and addiction counseling.

## Discussion

Despite the beneficial effects of bariatric surgeries on health, which includes reversal of type 2 diabetes mellitus and reduction in risk factors for heart disease, reports of substance use disorders after bariatric surgeries are on the rise [5-7]. Most studies looked at substance use after either Roux-en-Y gastric bypass or laparoscopic adjustable gastric band types of surgeries, which are the two most common bariatric surgeries in the United States [8]. The effects of various types of bariatric surgeries on subsequent substance use may be different. In our case, substance use disorder began one year after sleeve gastrectomy.

There have been multiple attempts to explain the increased risk of substance use disorders after bariatric surgeries. Similarities of the central nervous system response to food and drugs of abuse have been suggested [9]. Conditioned environmental cues, craving, and bingeing are phenomena occurring in both overeating and substance use disorders. McFadden suggested a “cross-addiction” model, in which the individual substitutes his addiction to food to substance use after the bariatric surgery [10]. Furthermore, there has been some evidence of altered alcohol metabolism after gastric bypass surgeries [11]. The marked reduction in weight could be a factor to consider, although a consistent association between low weight and the development of postoperative alcohol use disorder has not been found.

In the case presented, the patient had no history of substance use prior to the bariatric surgery. King et al. identified some factors that increase the likelihood of substance use after the surgery. These included male sex, young age, smoking, poor social support preoperatively, and undergoing Roux-en-Y gastric bypass surgery [3]. Psychological and social stressors, poor eating habits preoperatively, family history of substance use, as well as changes in the mechanisms of absorption after the surgery may play a role in the development of substance use disorder postoperatively [7].

Active alcohol or substance use is one of the contraindications to bariatric surgery according to many international guidelines. Therefore, it is important to thoroughly evaluate candidates for bariatric surgery for any history of prior or current substance use. Initial screening and recognition of patients at higher risk, with a consideration of family history and psycho-educational sessions about the risk of substance use postoperatively, has been recommended [6]. It is also necessary to closely follow up patients and intervene early if needed to reduce the risks related to substance use in this group of patients.

## Conclusions

This case report emphasizes that health care providers should assess the risks of substance use in candidates for weight reduction surgeries in order to improve the outcomes postoperatively. It appears that the risk of substance use disorder increases after different types of bariatric surgeries. Further research efforts to explore mechanisms linking bariatric surgery to substance use disorder and to identify specific risk factors are needed.
